# Sensory properties of hybrid composites based on poly(3,4-ethylenedioxythiophene)-porous silicon-carbon nanotubes

**DOI:** 10.1186/s11671-015-0896-1

**Published:** 2015-04-18

**Authors:** Igor B Olenych, Olena I Aksimentyeva, Liubomyr S Monastyrskii, Yulia Yu Horbenko, Lidia I Yarytska

**Affiliations:** Radioelectronics and Computer Systems Department, Ivan Franko National University of Lviv, 50 Dragomanov Street, 79005 Lviv, Ukraine; Physical and Colloidal Chemistry Department, Ivan Franko National University of Lviv, 6 Kyrylo and Mefodiy Street, 79005 Lviv, Ukraine; Thermodynamics and Physics Department, Lviv State University of Live Safety, 35 Kleparivska Street, 79000 Lviv, Ukraine

**Keywords:** Porous silicon, Poly(3,4-ethylenedioxythiophene), Carbon nanotubes, Composite film sensors, Sensing ability

## Abstract

**Abstract:**

In this work, we have prepared film sensor elements based on a hybrid system poly(3,4-ethylenedioxythiophene)-porous silicon nanocrystals-carbon nanotubes on flexible polymer substrates. Our FTIR spectroscopy-based studies for the molecular structure of the materials obtained suggest some interaction of their components in the hybrid layer. The influence of adsorption of water molecules on the conductivity and capacitance of the hybrid composites has been investigated in the temperature range of 20°C to 40°C. We have detected essential changes in the electrical conductivity and capacitance which depend on the humidity of the surrounding atmosphere. For estimating the sensing properties of our composites, we have analyzed the sensing abilities of the hybrid systems and their dynamic characteristics. The hybrid composites as working materials for the sensors provide improved performance of the latter. In particular, the response time is reduced by 3 to 5 times.

**PACS:**

73.63.-b, 73.61.Ph, 82.35.Np, 81.05.Rm

## Background

The development of flexible electronic, optical, and sensory devices requires novel composite materials which combine the properties of polymers and inorganic nanoparticles of different natures, such as semiconductor nanocrystals, metallic, silicon, and carbon nanoclusters [1-6]. Incorporation of low-dimensional components in composites is needed to maximize the size effects and large specific surface areas of nanoparticles.

It is known that conducting polymers with conjugated backbone and controlled electron characteristics represent promising components of organic–inorganic composites [7,8]. Novel organic materials for sensors and molecular electronics have been developed on the basis of those polymers [9-12]. The conducting polymers manifest electron delocalization arising from conjugated double bonds in a polymer backbone. They reveal electrical conductivity in the doped states (*p*- or *n*-doping) though remain insulators when undoped (a neutral state).

Protonic acids, electron donors (Na(naphthalide), Li, K, etc.), and electron acceptor substances (J_2_, Cl_2_, FeCl_3_, etc.) have been widely used as dopants [7-10]. Due to doping-dedoping processes, the electronic properties (e.g., the bandgap) of the conjugated polymers can be varied substantially [7-9]. Among the conducting polymers, poly(3,4-ethylenedioxythiophene) abbreviated hereafter as PEDOT is of particular interest owing to its remarkable optical, electrical, and electrochemical properties. To obtain PEDOT-based films deposited on almost any surfaces (e.g., conductive or dielectric, flexible, and polymeric), aqueous dispersion of polymeric complex of PEDOT doped by poly(styrenesulfonate) (PSS) is often used. A polymeric anion PSS acts simultaneously as an acid dopant and an anionic surfactant which stabilizes the dispersion of the polymer [13-15]. High enough processing ability and conductivity of the polymer complex PEDOT:PSS make it one of the most promising conducting polymers. Recently, it has been shown that adsorption of gas molecules such as CO [16] and NH_3_ [17], as well as vapors of organic solvents [15] or water molecules [18,19], can strongly affect different physical characteristics of PEDOT:PSS and the relevant composites. This suggests some ground for employing PEDOT:PSS in gas-sensing devices, e.g., humidity sensors.

Perhaps, one can use silicon and/or silica nanoparticles, which have high abilities to adsorb water molecules, for enhancing the performance of humidity sensors [1,20]. Silicon nanoclusters can be prepared using a straightforward procedure of electrochemical etching of single-crystalline silicon, with further formation of a layer of ‘porous silicon’ (PS). Ever since the intense visible photoluminescence of the PS has been detected at the room temperature by Canham in 1990 [21], the PS is regarded as a promising active element for optoelectronics. This includes applications associated with light-emitting diodes, phosphors, and optical transmission [22-24]. An essential point which limits the applications of PS in optoelectronics is its high surface sensitivity. On the one hand, this is a negative factor that imposes instability of the PS in air though, on the other hand, one can use this material for fabricating various sensors. An increase in the electrical resistance of the composites induced by silicon nanoparticles can be diminished basing on conductive admixtures such as carbon nanotubes (CNTs).

Notice that the CNTs are considered as a very promising material in the field of nanotechnology and sensor-related applications [5,17,25-27]. So, a network of single-walled CNTs shows good enough sensitivity to different gases (NO_2_ and NH_3_ [28,29] and vapors of organic solvents [28]). Multi-walled CNTs are sensitive to, e.g., H_2_ and CH_4_ [30]. They have already found applications in ammonia and water vapor gas sensors [31] due to larger effective surface areas, with many sites allowing the adsorption of the gas molecules.

We expect that the composites based on poly(3,4-ethylenedioxythiophene) combined with the PS nanocrystals and the CNTs should enhance the sensitivity and the operating speed of thin-film sensors fabricated with relatively simple and inexpensive technologies. As a consequence, the aim of the present work is to study the effect of adsorption of H_2_O molecules on the electrical parameters of film sensory systems based on hybrid composites PEDOT:PSS-PS-CNT.

## Methods

**Figure 1:**
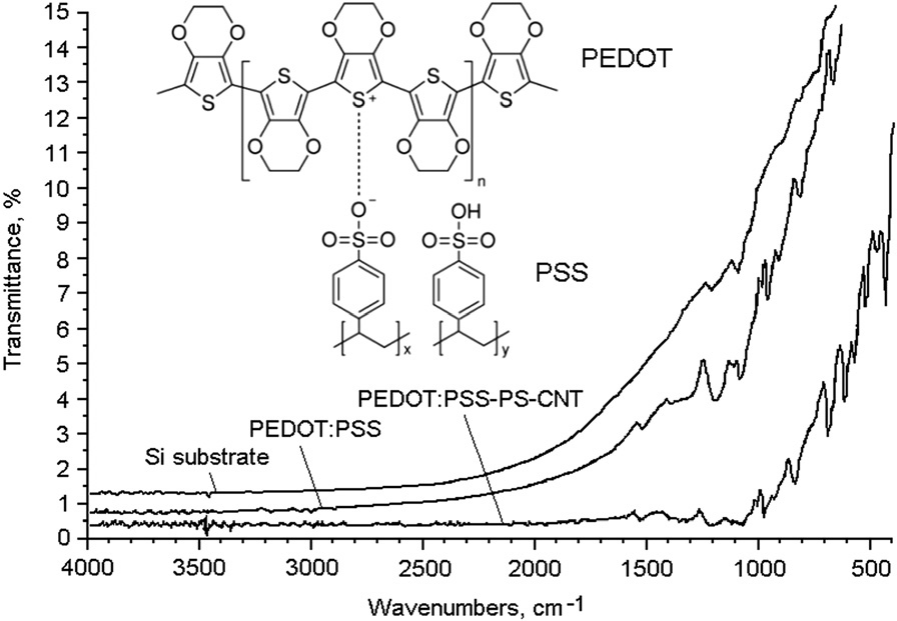
FTIR spectra of silicon substrate, PEDOT:PSS, and hybrid PEDOT:PSS-PS-CNT films on the silicon substrate. Inset shows chemical formula of PEDOT:PSS.

The hybrid composites were prepared from 1.5% aqueous polymer suspension PEDOT:PSS purchased from Sigma-Aldrich Co, St. Louis, MO, USA. Figure 1 (inset) shows a chemical formula of PEDOT:PSS. The other component of our hybrid composite was PS nanoparticles. The PS was manufactured by means of electrochemical etching performed in galvanostatic mode on single-crystalline silicon substrates, with had the typical thicknesses of 400 μm and the crystallographic orientation (100). The silicon substrates had the *n*-type conductivity (*n*-Si), with the specific resistance of 4.5 Ω cm. Ethanol solution of hydrofluoric acid (the volume ratio of the components HF:C_2_H_5_OH = 1:1) was used as an electrolyte. The anodic current (the density of 30 mA cm^−2^) passed during all the etching time (about 20 min). To ensure the availability of holes in the surface layer of *n*-Si, which were necessary for occurrence of anodic reactions and formation of the PS, the working surface of a silicon plate was irradiated with white light during the whole process of electrochemical etching. After cleaning our samples with distilled water and drying in air, a resulting porous layer has been taken off from the surface of the plate. It had the shape of a finely dispersed powder. The sizes of silicon particle ranged from a few tens of nanometers to several microns.

The third component of our composite was the multi-walled CNTs purchased from Sigma-Aldrich Co, St. Louis, MO, USA, with the diameters of 8 to 15 nm and the average length of 30 μm. The nanotubes were dispersed using ultrasonic processing in the mixture of nitric and sulfuric acids taken according to the ratio 3:1. The concentration of the CNTs was 0.5 mg per 1 ml. After multiple washing of the CNTs with distilled water, they were mixed with the nanoparticles of PS and the PEDOT:PSS solution and subjected to ultrasonic processing for 8 h. The suspension thus obtained was deposited onto a 0.4-mm-thick fluoroplastic substrate and then dried at the room temperature during 48 h. Eventually, a monolithic film of the hybrid PEDOT:PSS-PS-CNT nanocomposite was obtained. The thickness of the film was about 20 μm. The composite films PEDOT:PSS containing approximately 5% of CNTs and approximately 40% of PS were tested in our further experiments.

The molecular structure of the PEDOT:PSS-PS-CNT composite was explored using FTIR spectroscopy. The transmittance spectra were measured with an ‘Avatar’ spectrometer in the wave number region of 400 to 4,000 cm^−1^. To obtain the infrared (IR) spectra of the hybrid films, the composite PEDOT:PSS-PS-CNT was deposited, using the method described above, on a silicon wafer with the thickness of 400 μm. The absorption bands of the silicon substrate were easily identified (see [32]). To identify the absorption bands of the PEDOT:PSS-PS-CNT films, the literature data [13,32,33] may be considered.

For studying the sensory properties of the hybrid composite films, silver contacts were thermally deposited onto the surfaces of the films. The thickness of the contacts was about 0.5 μm. The adsorption processes in the hybrid PEDOT:PSS-PS-CNT films were studied in an airtight chamber, of which the gas medium can be changed. The water vapor concentration in the air was determined experimentally with a Honeywell HIH-4000-004 humidity sensor (Morristown, NJ, USA). The electrical parameters of our composite films were measured in the both DC and AC (1 kHz) regimes at two different temperatures, 20°С and 40°С.

## Results and discussion

To identify the components of the hybrid systems PEDOT:PSS-PS-CNT, we have measured their FTIR spectra. A comparative analysis of those spectra for the PEDOT:PSS system and the hybrid films deposited on the silicon substrates reveals a decreasing transmittance of the hybrid films, which has to be caused by the additional absorption and scattering of light by the nanotubes and the PS nanoparticles (see Figure 1).

The absorption bands located at 660 and 1,100 cm^−1^ correspond respectively to deformational Si-H mode and valence vibrations Si-O-Si of the silicon substrate (see [32]). In general, the bands observed are due to the oxidation of the silicon surface and adsorption of the water molecules from the atmosphere. The absorption bands observed in the FTIR spectra of the PEDOT:PSS-PS-CNT composite, which are located in the regions 1,080 to 1,200 and 1,500 to 1,550 cm^−1^, are characteristic for С-О-С complexes and С = С, С-С vibrations of thiophene rings [13,33]. The absorption band at 700 cm^−1^ can be ascribed to valence vibrations С-S. The band with the maximum located at 860 cm^−1^ and the peaks in the region of 950 to 1,000 cm^−1^ may originate from deformational vibrations Si-OH and С-Н, respectively [13]. A new band at 470 to 520 cm^−1^ can appear due to the carbon bonds available in the CNTs.

Note that the hybrid PEDOT:PSS-PS-CNT films show more intense absorption bands, as compared to the polymer. Moreover, they manifest different absorption profiles in the regions of 620 to 800 cm^−1^ and 1,300 to 1,420 cm^−1^. This can be readily explained by the interactions among the structural parts of the polymer and the CNTs, and the PS surface. In the spectral interval 3,400 to 4,000 cm^−1^, we have observed the absorption bands induced by the hydroxyl groups from Si-OH and by the adsorbed water molecules. It is worthwhile noting that the hydrophilic properties of the hybrid composites PEDOT:PSS-PS-CNT expand their prospects as working elements for the humidity sensors.

**Figure 2:**
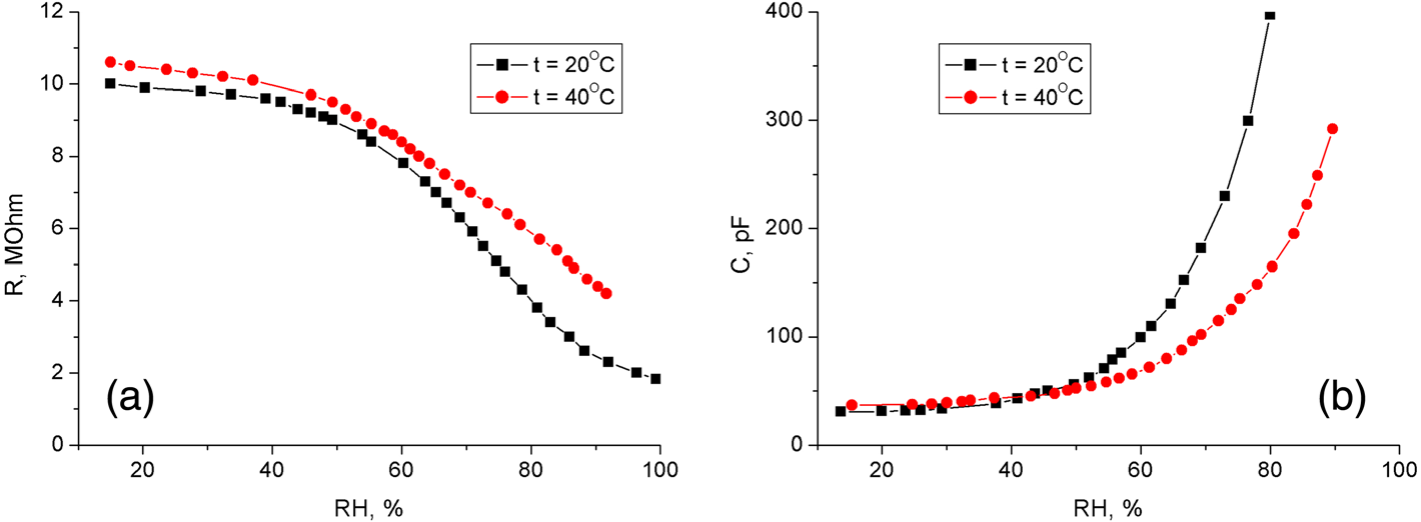
Resistance **(а)** and capacitance **(b)** of PEDOT:PSS-PS-CNT films as functions of the relative air humidity.

We have found experimentally that the electrical characteristics of our thin-film sensor elements are strongly dependent on the surrounding atmosphere. In particular, the increasing relative humidity results in significant decrease of the electrical resistance and increase of the capacitance of the hybrid PEDOT:PSS-PS-CNT films (see Figure 2).

The dependences mentioned above can be explained by the interaction of water molecules with the surface of the composite, which leads to changing electric parameters of the PS nanocrystals (see [20]). As a result, one observes increasing conductivity of the hybrid film. On the other hand, water impurities might induce additional (or ‘secondary’) doping of the conjugated polymer PEDOT:PSS. This manifests itself in change of the chain shape to an ‘unfolded spiral’ and, therefore, stimulates increase in the conductivity [15].

Besides the influence on the electronic structure of silicon nanocrystals, the effective dielectric permittivity of the hybrid film also varies due to the adsorption of the water molecules, which have large dielectric constant (*ε* = 81) as compared to the silicon. This is why increasing relative humidity of the air causes a noticeable increase of the capacitance of the hybrid nanocomposites PEDOT:PSS-PS-CNT.

An important factor of studying the mechanisms of variation of the physical parameters of sensor materials under adsorption-desorption interactions with gaseous media is the determination of the sensing ability of the material. To estimate the sensing (i.e., gas-sensitive) properties of the hybrid composite PEDOT:PSS-PS-CNT films, we have calculated their ‘sensing ability’ using the known relation [34] ${\gamma}_G=\frac{1}{S}\frac{\varDelta S}{\varDelta p}$.

**Figure 3:**
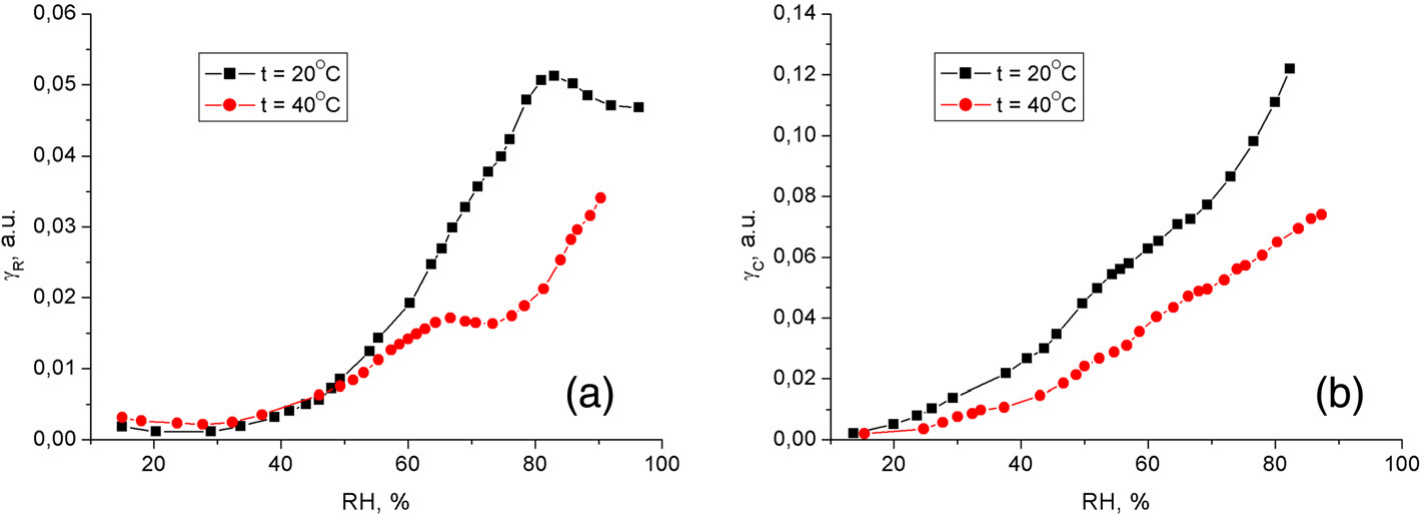
Sensing abilities of resistive **(а)** and capacitive **(b)** sensors based on the PEDOT:PSS-PS-CNT films as functions of the relative air humidity.

Here Δ*S*/*S* denotes the relative change in the electrical resistance or the capacitance of the structure, and Δ*p* is the change in the relative air humidity. The calculated dependences of sensitivity for the two different film sensor elements on the humidity are shown in Figure 3.

The sensory properties of the composite film are better within the humidity region 80% to 100%. Such a behavior observed at large concentrations of the water vapor, when the pores of the nanocomposite contain a significant amount of condensed water, might be related to the change in the water adsorption mechanism (from monomolecular to polymolecular). The latter mechanism is mainly governed by the intermolecular interactions inside the film [35,36]. The ambient temperature also affects the electrical resistance and the capacitance of the sensing elements. Although the increase in the temperature up to 40°С does not change a general character of the dependences, it decreases the sensitivity of our structures to the water vapor concentration variations.

**Figure 4:**
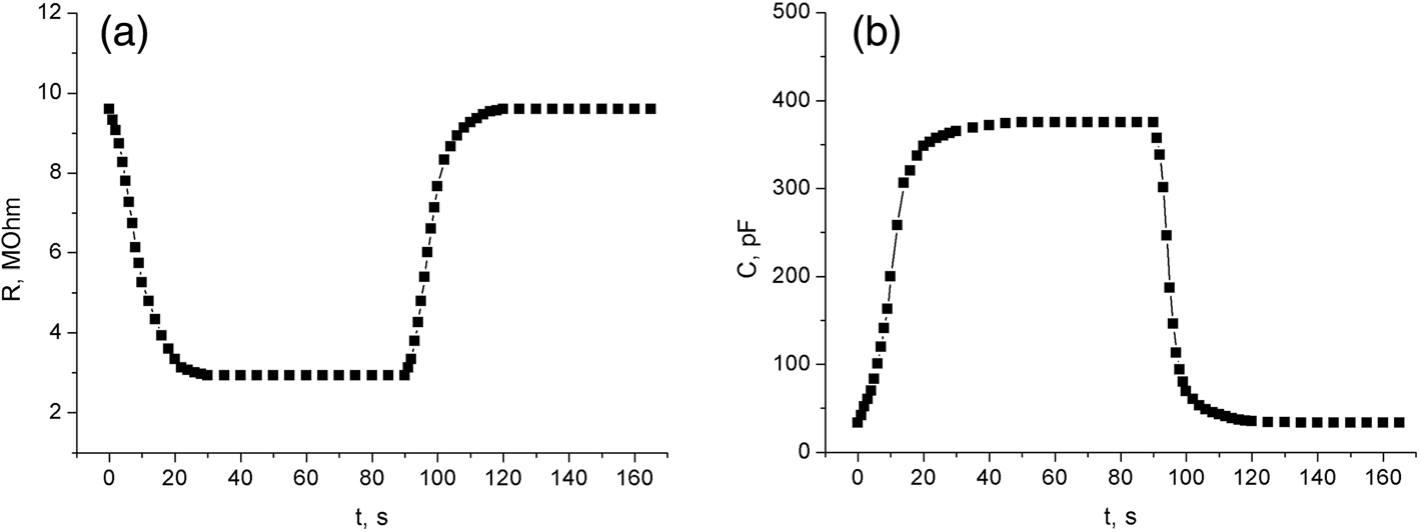
Responses of resistance **(a)** and capacitance **(b)** of the sensors based on PEDOT:PSS-PS-CNT films to the pulse of relative air humidity.

The kinetics of the sensor response to varying relative air humidity has a two-stage shape. The dynamic dependences for the sensor elements based on the PEDOT:PSS-PS-CNT film are shown in Figure 4.

The interaction with the water vapor has a character of physical adsorption. It represents an inverse process with low activation threshold. This hypothesis is substantiated by the fact that the initial conductivity and capacitance of the hybrid films are restored after scavenging and pumping off the water vapor from the experimental chamber. The time of response of the sensory elements to changing gas concentration is about 30 s, which is quite sufficient for microelectronic humidity sensors. When compared with the moisture-sensitive structures PS-silicon substrate [20], the response of our hybrid film sensors is much more (3 to 5 times) faster.

## Conclusions

We have created flexible sensory elements based on the PEDOT:PSS-PS-CNT composite films. Using the FTIR spectroscopy, we have determined the components present in the hybrid layer. In particular, we have found the main IR absorption bands related to the structural chains of the polymer. The changes in the IR absorption observed by us may be due to some interactions of the polymer complexes with the PS nanocrystals and the CNT, as well as due to the adsorption of water molecules and hydroxyl groups.

It has been revealed experimentally that the adsorption of water molecules changes the electrophysical properties of the composites under test. The analyses of dependences of the electrical conductivity, the capacitance, and the sensing ability on the concentration of water vapor point to a notable increase in the sensitivity of the composite at the relative humidity approximately 80%. This may be associated with changing conductivity mechanisms. The kinetics of the response of the hybrid composites PEDOT:PSS-PS-CNT to the changing water vapor concentration is fast enough to be employed in various microelectronic humidity sensors. Finally, the results obtained in the present study would allow further optimization of flexible film sensors.
